# Successful Treatment for a Huge Pulmonary Pseudoaneurysm in the Interlobar Segment with Coil Embolization: A Case Report

**DOI:** 10.3400/avd.cr.25-00132

**Published:** 2026-02-17

**Authors:** Masao Takahashi, Koichiro Matsuura, Ken Nakazawa, Yoko Usami, Shunsuke Yamada, Satoru Mochida, Cho Konjo, Kaiji Inoue, Eito Kozawa

**Affiliations:** 1 Department of Diagnostic Radiology, Saitama Medical University Hospital, Iruma, Saitama, Japan; 2 Department of Diagnostic Radiology, Saitama Medical University International Medical Center, Hidaka, Saitama, Japan; 3 Department of Gastroenterology and Hepatology, Saitama Medical University Hospital, Iruma, Saitama, Japan; 4 Department of Respiratory Medicine, Saitama Medical University Hospital, Iruma, Saitama, Japan

**Keywords:** pulmonary artery pseudoaneurysm, coil embolization, endovascular therapy

## Abstract

A 62-year-old male with empyema underwent an attempted pleural drainage, which resulted in iatrogenic pseudoaneurysm formation of the right pulmonary artery in the interlobar segment. Endovascular treatment was favored over surgical intervention due to the presence of empyema. The pulmonary artery in the interlobar segment was embolized with metallic coils, with inevitable occlusion of blood perfusion in the middle and lower lobes. Despite the perfusion loss in a large lung territory, the patient eventually required no supplemental oxygenation. Coil embolization can be a favorable alternative to stent-grafting for pseudoaneurysm even in the interlobar segment, despite the potential risk of post-procedure hypoxemia.

## Introduction

Pulmonary artery (PA) injury is a rare but life-threatening condition that can cause severe hypoxemia from massive hemoptysis and hemodynamic instability due to massive hemothorax. Hemorrhage from PA injury in peripheral branches can be treated with endovascular embolization without much concern.^1,2)^ However, in cases of proximal PA injury, embolization is typically avoided due to the risk of losing pulmonary arterial perfusion to a large area. In such cases, a surgical approach, like vascular repair and lung resection procedures, remains the more common hemostatic treatment.^3)^ The interlobar segment of the PA is located distal to the proximal PA segment and gives rise to multiple branches supplying the superior, middle, and lower lobes. Injury to this segment is very rare and has been reported in association with surgery, PA catheter, trauma, and tumor involvement.^4)^

We report a case of PA injury in the interlobar segment that was successfully treated with coil embolization, highlighting a nonsurgical approach for proximal PA injury.

## Case Report

A 62-year-old male with advanced hepatocellular carcinoma underwent an attempted pleural drainage for empyema secondary to tumor rupture into the right thoracic cavity. During the insertion of a 22-Fr chest tube using the trocar technique, approximately 500 mL of blood rapidly drained through the placed tube and continued persistently. The patient also complained of sudden-onset mild hemoptysis following tube placement. The tube was immediately removed, with no signs of hemodynamic instability except persistent hemoptysis.

**Fig. 1:**
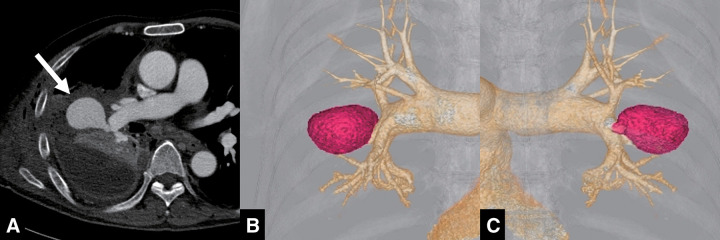
CT pulmonary angiography before embolization. (**A**) The pseudoaneurysm (white arrow), surrounded by empyema, extended from the interlobar segment of the right pulmonary artery. (**B**) Three-dimensional angiographic reconstruction (anterior–posterior view) revealed the pseudoaneurysm (red region) arising from the interlobar segment of the right pulmonary artery. (**C**) Three-dimensional angiographic reconstruction (posterior–anterior view). CT: computed tomography

Initially, the drained fluid was interpreted as empyema contents mixed with old blood. However, the development of chest discomfort and worsening anemia on laboratory tests prompted further investigation. Contrast-enhanced computed tomography (CT) performed the next day revealed a huge pseudoaneurysm of the right PA in the interlobar segment between the superior and inferior lobe branches (**Fig. 1A–1C**). The pseudoaneurysm was encased by empyema, which prevented active bleeding into the thoracic cavity. This case was diagnosed as an iatrogenic PA injury caused by the chest tube insertion.

The patient was initially referred to the Department of Thoracic Surgery, but the presence of empyema discouraged emergent open thoracotomy for hemostasis. He was subsequently referred to the Department of Interventional Radiology, because an endovascular approach was favored over surgical intervention for hemostatic management.

**Fig. 2:**
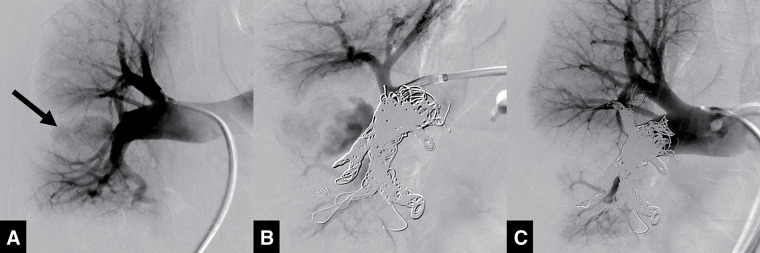
Digital subtraction angiography during the embolization procedure. (**A**) Right pulmonary artery angiography demonstrated jet-like contrast flow into the pseudoaneurysm extending from the interlobar segment (black arrow). (**B**) Metallic coils were deployed from the middle and lower lobe branches to the level of the A6 segmental branch, but the contrast filling remained in the pseudoaneurysm. (**C**) Additional coils were deployed up to the level of the superior lobe trunk, resulting in complete exclusion of the pseudoaneurysm.

An endovascular approach was finally selected for hemostatic treatment of the PA pseudoaneurysm. 8-Fr guiding sheaths were inserted into both femoral veins to access the right PA. Right pulmonary angiography showed a huge pseudoaneurysm extending laterally from the right PA interlobar segment between the superior and inferior lobe branches (**Fig. 2A**). Based on the PA branch anatomy and pseudoaneurysm location, coil embolization in the interlobar segment was selected to achieve exclusion of the pseudoaneurysm for hemostasis.

A 6-Fr balloon catheter was positioned in the proximal segment of the right PA to ensure temporary blood flow occlusion in case of massive hemorrhage or hemoptysis during the embolization procedure. Metallic coils were initially deployed in the branches of the middle and inferior lobes, followed by additional coils placed in the interlobar segment to occlude the entry of the pseudoaneurysm. When the proximal edge of the coil mass reached the level of the superior segmental branch of the lower lobe (A6 segmental branch), PA angiography showed residual filling of the pseudoaneurysm (**Fig. 2B**). Further coils were subsequently placed up to the level of the superior trunk; then, PA angiography confirmed complete exclusion of the pseudoaneurysm (**Fig. 2C**). Given the anticipated need for a large number of coils due to the huge vascular volume, coils with high packing density and strong embolization ability were selected to ensure efficient vessel occlusion. A total of 26 coils were used for embolization, including 19 Interlock-35 (Boston Scientific, Marlborough, MA, USA) and 7 Target XXL (Stryker, Kalamazoo, MI, USA).

**Fig. 3:**
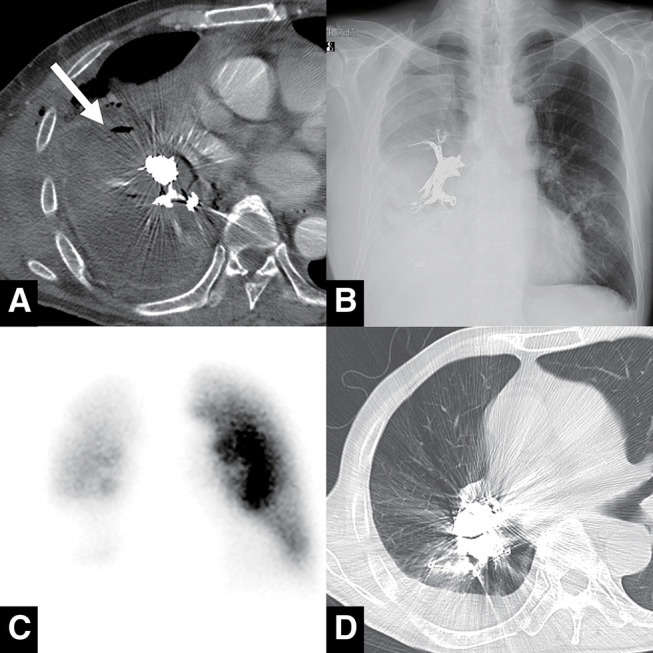
Follow-up imaging after embolization. (**A**) Contrast-enhanced CT performed 2 days post-procedure demonstrated no contrast filling in the pseudoaneurysm (white arrow). (**B**) Chest X-ray on the following day showed a large coil mass extending from the middle and lower lobe branches to the interlobar segment. (**C**) Ventilation–perfusion scan revealed perfusion defects in the right middle and lower lobes. (**D**) Non-contrast CT obtained 2 months later demonstrated complete disappearance of the pseudoaneurysm with resolution of empyema. CT: computed tomography

The patient was carefully monitored after the embolization procedure to check for early signs of rebleeding and hypoxia. Hemoptysis resolved on post-embolization day 1, and contrast-enhanced CT performed on day 2 demonstrated no contrast filling of the pseudoaneurysm (**Fig. 3A**). Chest X-ray demonstrated a coil mass in the right PA from the middle and lower lobe branches to the interlobar segment (**Fig. 3B**). Lung ventilation–perfusion (V/Q) scanning revealed a perfusion mismatch in the right middle and lower lobes, consistent with coil-induced flow occlusion (**Fig. 3C**). Despite the loss of perfusion in these lobes, the patient required no additional oxygen supplementation compared to his pre-embolization status, maintaining an oxygen saturation of 95% with 2 L/min of oxygen via nasal cannula. Several days after the embolization, purulent sputum with old blood began to be expectorated, indicating the formation of a fistula between the empyema cavity and the airway. On post-embolization day 11, he was weaned off supplemental oxygen without exertional dyspnea, with a stable oxygen saturation of 96%. Approximately 1 month after embolization, he was discharged from the hospital by walking independently without any need for home oxygen therapy. Follow-up CT performed 2 months after embolization demonstrated no recurrence of the pseudoaneurysm and almost total resolution of empyema with conservative management alone through spontaneous drainage via the airway (**Fig. 3D**).

## Discussion

PA pseudoaneurysm is generally caused by infection, tumor erosion, or iatrogenic injury such as biopsy, chest tube insertion, or catheter placement like Swan–Ganz catheter. Although PA pseudoaneurysm is not a frequent clinical condition, it often results in life-threatening complications like massive hemoptysis and hemothorax. When PA pseudoaneurysm is located in peripheral PA branches, embolization is effective as the first line treatment due to lower invasiveness compared with surgical intervention, involving only minimal reduction in PA perfusion.^1)^ In contrast, embolization for proximal PA segment is clinically challenging because it inevitably requires sacrificing a broad area of PA perfusion.

Endovascular therapy for PA pseudoaneurysm possibly includes several alternative techniques, such as stent-graft placement,^5)^ occlusion of the pseudoaneurysm entrance with a vascular plug,^6)^ and filling of the pseudoaneurysm sac with embolic materials.^7,8)^ Stent graft may be the most favorable option because the pseudoaneurysm can be excluded without loss of parent arterial flow. In the present case, however, this approach was not feasible due to an insufficient landing zone to achieve complete sealing of the involved PA segment. The pseudoaneurysm neck was located near the bifurcation of multiple lower lobe segmental arteries, where the vascular diameter transitioned from a large trunk to smaller branches. Additionally, the presence of multiple bifurcations beyond the aneurysmal neck led to technical difficulty in complete sealing of the distal landing zone with a stent graft, given its simple tubular device structure. This anatomical-device mismatch made it difficult to achieve adequate proximal and distal landing zones. Consequently, in the present case, simple coil embolization was finally selected as a more easily accessible and technically straightforward option because stent-grafting was not well-suited to the complex anatomy of the PA interlobar segment.

Placement of a vascular plug across the pseudoaneurysm entrance may effectively block inflow, but this technique requires advancement of a large-bore guiding sheath to the pseudoaneurysm, which carries the risk of aneurysmal rupture during manipulation. Filling the pseudoaneurysm sac with embolic materials, such as metallic coils or liquid materials like thrombin and glue, represents another therapeutic option. However, this method is often associated with incomplete hemostasis and recurrence.^9)^ Additionally, the size of the pseudoaneurysm in the present case was so large that complete filling may have been difficult to achieve stable hemostasis.

Placement of endovascular devices, such as coils, stent grafts, and vascular plugs, raises concern about device-related infection when these materials are deployed adjacent to infectious lesions. From this perspective, the use of embolic agents, such as gelatin sponge or liquid embolic materials, may provide more favorable long-term safety. In the present case, however, these embolic agents were unlikely to achieve effective embolization because the huge vascular volume of the PA required significantly large amounts of them for complete hemostasis. Furthermore, injection of large amounts of such embolic agents is likely to trigger significant inflammatory reactions, potentially exacerbating the existing pleural empyema.

In general, coil embolization in the parent artery can definitively occlude blood flow to a pseudoaneurysm when both inflow and outflow vessels are occluded (sandwich technique).^10)^ In the case of PA pseudoaneurysm, coil embolization in the parent artery inevitably involves sacrificing pulmonary perfusion. Embolization of the interlobar segment, in particular, results in loss of perfusion to a large vascular territory, including the middle and lower lobes. This perfusion loss leads to V/Q mismatch, resulting in increased alveolar dead space, where alveoli are ventilated but not perfused for gas exchange. In the present case, the patient finally did not require long-term supplemental oxygen, likely because there was no pre-existing pulmonary impairment. However, the potential need for introduction of lifelong oxygenation therapy following embolization should be carefully considered prior to the procedure. Despite the inevitable risk of hypoxemia, coil embolization remains a definitive and technically straightforward hemostatic method for PA pseudoaneurysm, even when located in the interlobar segment, particularly in emergency situations.

Embolization of a large PA territory is sometimes associated with ischemic pleural symptoms, such as chest pain. In the present case, such ischemic change could have adversely affected the healing process of empyema. Meanwhile, the presence of inflammatory lesions within the pleural cavity increases systemic arterial flow from arteries such as the bronchial and intercostal arteries. This augmented systemic blood flow may have partially compensated for the reduced PA flow in this case.

## Conclusion

PA pseudoaneurysm in the interlobar segment can be treated with coil embolization, despite the potential requirement for introducing supplemental oxygen therapy. The treatment strategy for PA pseudoaneurysm should be decided through multidisciplinary discussion.
